# Ethanol Induction of Innate Immune Signals Across BV2 Microglia and SH-SY5Y Neuroblastoma Involves Induction of IL-4 and IL-13

**DOI:** 10.3390/brainsci9090228

**Published:** 2019-09-10

**Authors:** Colleen J. Lawrimore, Leon G. Coleman, Jian Zou, Fulton T. Crews

**Affiliations:** 1 Bowles Center for Alcohol Studies, School of Medicine, University of North Carolina at Chapel Hill, Chapel Hill, NC 27599, USA; 2 Curriculum in Neurobiology, University of North Carolina at Chapel Hill, Chapel Hill, NC 27599, USA; 3 Department of Pharmacology, School of Medicine, University of North Carolina at Chapel Hill, Chapel Hill, NC 27599, USA; 4 Department of Psychiatry, School of Medicine, University of North Carolina at Chapel Hill, Chapel Hill, NC 27599, USA

**Keywords:** microglia, neurons, ethanol, innate immune, co-culture

## Abstract

Innate immune signaling molecules, such as Toll-like receptors (TLRs), cytokines and transcription factor NFκB, are increased in post-mortem human alcoholic brain and may play roles in alcohol dependence and neurodegeneration. Innate immune signaling involves microglia -neuronal signaling which while poorly understood, may impact learning and memory. To investigate mechanisms of ethanol induction of innate immune signaling within and between brain cells, we studied immortalized BV2 microglia and SH-SY5Y human neuroblastoma to model microglial and neuronal signaling. Cells were treated alone or in co-culture using a Transwell system, which allows transfer of soluble mediators. We determined immune signaling mRNA using real-time polymerase chain reaction. Ethanol induced innate immune genes in both BV2 and SH-SY5Y cultured alone, with co-culture altering gene expression at baseline and following ethanol exposure. Co-culture blunted ethanol-induced high mobility group box protein 1 (HMGB1)-TLR responses, corresponding with reduced ethanol induction of several proinflammatory NFκB target genes. In contrast, co-culture resulted in ethanol upregulation of cytokines IL-4 and IL-13 in BV2 and corresponding receptors, that is, IL-4 and IL-13 receptors, in SH-SY5Y, suggesting induction of a novel signaling pathway. Co-culture reduction in HMGB1-TLR levels occurs in parallel with reduced proinflammatory gene induction and increased IL-4 and IL-13 ligands and receptors. Findings from these immortalized and tumor-derived cell lines could provide insight into microglial-neuronal interactions via release of soluble mediators *in vivo*.

## 1. Introduction:

Alcohol causes a dysregulation of the neuroimmune system in the brain which corresponds to alcohol-induced neurodegeneration and addiction pathology [1,2,3,4]. Alcohol consumption is also correlated with a variety of negative health outcomes, including but not limited to onset of alcohol use disorders (AUD), fetal alcohol spectrum disorders, alcoholic liver disease and cancers. In particular, Toll-like receptor (TLR) signaling has been suggested to be implicated in alcohol pathology. In the periphery, TLRs respond to viral and bacterial components, whereas in brain they respond to endogenous agonists such as high mobility group box protein-1 (HMGB1), which activates multiple TLRs [5,6,7]. Activation of TLRs leads to stimulation of transcription factors like NFκB, which regulates transcription of cytokines, propagating inflammation through their respective receptors [8]. Studies by Consuelo Guerri laid the groundwork for studying TLRs in alcohol pathology, finding a crucial role for TLR4 in ethanol-induced immune responses and neurodegeneration [9,10,11,12]. Multiple components of the TLR pathway have since been found to be upregulated in post-mortem human alcoholic brain; in particular, there is increased expression of TLR3, TLR4, TLR7 [13,14], as well as phosphorylated (activated) NFκB [15] and cytokines such as IL-1β and HMGB1 [13,16,17]. Other components of the TLR signaling, including TLR4 adapter MD-2 and downstream TLR signaling kinase TBK1, have been implicated in alcohol pathology [11,18]. Furthermore, alcohol-potentiated increases in TLR3 and TLR4 increase TLR agonist-induction of cytokines [19,20]. NFκB-related cytokines, such as MCP1 and its receptor, CCR2, have also been implicated in ethanol drinking [21]. These data therefore suggest that TLR pathways are implicated in alcohol pathology but exact mechanisms remain to be elucidated, such as the respective involvement of various cell types as well as cell-to-cell interactions that bring about ethanol-induced innate immune signaling. 

The innate immune system takes on unique roles outside of traditional inflammation mediation in brain. In particular, cytokines often function as signaling molecules between different cell types in brain. Several of these systems have been previously described for their roles in development and synaptic pruning, such as neuronal fractalkine (CX3CL1) signaling through its microglial receptor (CX3CR1) or the complement system [22,23]. Cytokines released by activated microglia can also cause neurotoxic activity in astrocytes [24]. However, the ability of other cytokine systems to function as microglial-neuronal signaling mechanisms is less well described. In particular, IL-4 and IL-13 signaling is an emerging glia-to-neuron signaling pathway. In the periphery, IL-4 and IL-13 function as anti-inflammatory cytokines produced by Th2 cells [25]. The receptors for these cytokines form a heterodimer, consisting of IL-4Rα1 (IL-4R) and IL-13Rα1 (IL-13R), indicating that these two cytokines have similar signaling pathways. In brain, microglia appear to express low levels of IL-4 and IL-13 basally but can induce expression under certain conditions [26,27]. Interestingly, IL-13 receptor is located on dopaminergic neurons and overactivation of this receptor has been linked to loss of dopaminergic cells [28], prompting studies on the impact of alcohol exposure due to the importance of dopamine for drug-induced reward. Furthermore, IL-4 and IL-13 also have roles in learning and memory [29,30]. Stimulation of this pathway leads to activation of transcription factor STAT6, which is linked to IL-10 and TGFβ expression, cytokines which themselves have roles in neuroprotection and learning/memory [31,32,33,34].

Microglia, the canonical macrophages in brain, are thought to be the main mediator of innate immune signaling in brain and are therefore the focus of many studies on ethanol-induced innate immune signaling. Indeed, Guerri’s previous studies have shown that ethanol induces various cytokines in microglial cultures [10,12]. Our lab has reported neuronal localization of TLR3, TLR4 and HMGB1 in rat brain [35], as well as neuronal IL-1β in mouse brain [17]. In addition, we recently found that ethanol increases both TLR3 and TLR7 in SH-SY5Y neuroblastoma and at lower ethanol concentrations than in BV2 microglia, in addition to HMGB1 release in both cell types [36]. These experiments were conducted in single cell types, whereas in brain there is constant and dynamic communication between neurons and microglia [37]. However, it remains unknown how microglial-neuronal interactions via secretion of soluble mediators can affect these ethanol-induced changes in innate immune gene expression. In addition, while IL-13 is increased in serum of cirrhotic alcoholic patients [38], little is known about how alcohol affects IL-4 and IL-13 signaling.

In this study, we examine how co-culture of BV2 microglia and SH-SY5Y neuroblastoma influences ethanol-induced innate immune signaling of these cell lines. Co-culture modules using glial cells and SH-SY5Y have been used previously in multiple disease settings [39,40]. Since SH-SY5Y adopt a dopaminergic phenotype [41] and IL-4 and IL-13 modulate dopaminergic signaling, SH-SY5Y could provide insight into responses of dopaminergic neurons. We extend previous findings by finding that co-culture modifies induction of cytokines and TLRs in BV2 and SH-SY5Y. We next found that ethanol upregulates IL-4 and IL-13 signaling in co-cultured BV2 and SH-SY5Y. Using a primary hippocampal-entorhinal slice culture model we found that IL-4 and IL-13 diminish expression of pro-inflammatory cytokines TNFα and IL-1β. Thus, co-culture of BV2 and SH-SY5Y results in differential immune responses to ethanol than exposure of each cell type to ethanol alone. 

## 2. Methods

### 2.1. Cell Lines and Treatment

The immortalized mouse microglial cell line, BV2, were acquired from ICLC (Genoa, Italy, #ATL03001). BV2 were cultured using Dubecco’s modified Eagle serum (DMEM, Life Technologies #11995-065, Carlsbad, CA) supplemented with 10% fetal bovine serum (FBS, Life Technologies #26140-079), 1X GlutaMAX (Life Technologies #35050-061) and 1X antibiotic-antimycotic (Life Technologies #15240-062). In the BV2 alone group, media was changed to 2% FBS approximately 16 h prior to treatment.

The human neuroblastoma-derived cell line SH-SY5Y were acquired from ATCC (Manassas, Virginia, #CRL-2266). SH-SY5Y were cultured using DMEM/F-12 + GlutaMAX (Life Technologies #10565-018), 10% FBS and 1X antibiotic-antimycotic. Prior to treatments, SH-SY5Y were differentiated using 10 uM retinoic acid (RA, Sigma-Aldrich #R2625, St. Louis, MO,) for 4 days in Neurobasal media (Life Technologies #21103-048) containing 2% B27 media supplement (Life Technologies #17504-044), 0.5 mM GlutaMAX and 1X antibiotic-antimycotic. In the SH-SY5Y alone group, media was refreshed 16 h prior to treatment.

In all groups treated with ethanol, a concentration of 100 mM (equivalent to 460 mg/dL or 0.46%) for 24 h was used. This high concentration is consistent with the high (>400 mg/dL) blood alcohol concentrations (BACs) achieved in alcoholics in emergency rooms [42], including awake and conscious patients with a BAC of greater than 500 mg/dL [43]. Binge models in rodents that achieve similarly high BACs have also been used to model heavy drinking [44]. Cells exposed to ethanol were placed into an ethanol-saturated chamber using a beaker with 200 mL of 4% ethanol, allowing the media to remain constant throughout the duration of the experiment as we have reported previously [45]. Control groups were given an equal volume of vehicle (PBS). Following treatment, cells lysates were harvested for real-time polymerase chain reaction (RT-PCR) analysis. For each experiment, 5-6 culture wells were treated per group. 

### 2.2. Transwell Co-Culture Model

**Figure 1:**
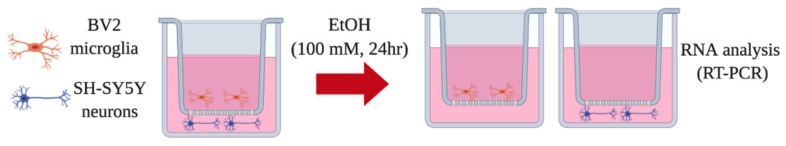
Transwell co-culture model for examining ethanol-induced changes in BV2 microglia and SH-SY5Y.

SH-SY5Y were plated (3.75 × 10^5^ cells per well) on 6-well plates. After the differentiation procedure described above, media was refreshed to 10% FBS-containing DMEM and Transwell inserts (Corning CLS3452, Corning, NY, USA) were placed on top of the SH-SY5Y-containing wells and allowed to equilibrate for 30 minutes. Transwells are a well-characterized 3D model of co-culture and have been used extensively for neuronal co-cultures [40,46,47,48], including with SH-SY5Y and BV2 in particular [49]. BV2 were plated (1.5 × 10^5^ cells per well) on top of the Transwell inserts; see Figure 1 for a schematic. Sixteen hours prior to ethanol treatment (30 h following plating of BV2), media was changed to 2% FBS. See Figure 1 for a diagram of the treatment outline.

SH-SY5Y were plated on the bottom of 6-well culture plates and Transwell inserts were placed in the wells. BV2 microglia were plated on top of the Transwells. Cells were treated with ethanol (100 mM) for 24 h, keeping the ethanol concentration constant by saturated the chamber with ethanol. This was followed by RNA isolation in both BV2 microglia and SH-SY5Y neuron cell lysates.

### 2.3. Hippocampal Entorhinal (HEC) Slice Culture

All protocols followed in this study were approved by the Institutional Animal Care and Use Committee at UNC and were in accordance with National Institutes of Health regulations for the care and use of animals in research. Organotypic brain slice cultures were prepared as described previously [50]. Briefly, the hippocampal entorhinal region was dissected and sliced transversely (375 μm thick) from postnatal day 7 rat pups. HEC slices were placed onto tissue insert membrane (10 slices/insert) and cultured with medium containing 75% MEM with 25 mM HEPES and Hank’s salts, 25% horse serum (HS), 5.5 g/L glucose, 2 mM L-glutamine in a humidified 5% CO2 incubator at 36.5 °C for 7 days in vitro (DIV), followed by 4 DIV in medium containing 12.5% HS and then 3 DIV in serum-free medium supplemented with N2 (Thermo Fisher #17502001, Waltham, MA, USA). The cultures after 14 DIV were used for experiments and drug treatments with serum-free N2- supplemented medium. For ethanol exposures, slices were exposed to ethanol (100 mM) for 48 h in a desiccator saturated with an equivalent concentration of ethanol to prevent evaporation from the media. Recombinant rat IL-4 protein (500 ng/mL; R&D Systems, #504-RL-005) and recombinant rat IL-13 protein (1 ug/mL; R&D Systems, #1945-RL-025) were also added to the indicated groups for 48 h.

### 2.4. Real-Time (RT)-PCR

RNA was extracted from cell lysates using TRIzol (Invitrogen #15596026, Carlsbad, CA, USA). RNA was extracted from HEC slices using RNeasy Mini Kit (Qiagen #74104, Hilden, Germany). RNA concentration was determined using a Nanodrop (Thermo Fisher) and was reverse-transcribed to cDNA. The SYBR green PCR master mix (Life Technologies #4309155) was used for real-time PCR analysis. The relative differences in expression between groups were expressed using cycle time (Ct) values normalized with β-actin and relative differences between control and treatment groups were calculated and expressed as relative increases setting control as 100%. Primers used are listed in Table 1.

### 2.5. Enzyme-Linked Immunosorbent Assay (Elisa)

Media (1 mL) was collected after ethanol treatment and spun at 500× g for 10 minutes to remove cell debris. HMGB1 protein concentration in media was determined using an HMGB1 ELISA kit (IBL, Hamburg, Germany), per manufacturer’s directions. Briefly, samples were diluted 1:3 using the supplied Diluent buffer. The HMGB1 standards (0–10 ng/mL) were prepared using the supplied HMGB1 stock and Diluent Buffer. 50 uL of Diluent buffer was added to each well of the supplied 96 well plate (which came pre-coated with an anti-HMGB1 antibody) using a multi pipette, followed by 50 uL of each standard and sample, both in duplicate. The plate was then incubated at 37 °C for approximately 24 h. The wells were then aspirated and the plate was washed 5 times with the supplied Wash Buffer (1×). 100 uL of the supplied Enzyme conjugate was added to each well and incubated at 25 °C for 2 h. The plate was then washed 5 times with Wash Buffer and 100 uL of the supplied Color solution was added to each well. Following a 30-minute incubation at room temperature, 100 uL of the supplied Stop solution was added into each well. The optical density was measured using a spectrophotometer at 450 nm, with a reference wavelength at 600. To calculate HMGB1 concentration, each duplicate was first averaged followed by subtraction of the blank value (i.e., only Diluent Buffer) to remove background. The OD of the standards (x-axis, log scale) was plotted against the concentration (y-axis, log scale), followed by a linear trendline. The concentration of each sample was determined according to the trendline equation, followed by correction based on the dilution factor.

### 2.6. Statistical Analysis

Two-way ANOVAs were performed on all co-culture experiments (consisting of 5–6 wells in each group) to determine the main effect and interaction of co-culture and ethanol treatment. The means represent the mean of the values from the 5–6 wells assessed for each group. Post-hoc Bonferroni analyses were used to determine significant effects between groups (co-culture control, co-culture ethanol, alone-control and alone-ethanol) [51]. HEC slice culture data was analyzed using a one-way ANOVA followed by post-hoc Bonferroni’s test. Standard error of the mean (SEM) bars are shown for the data in order to better show how precisely the data define the mean in each particular group [52]. A *p*-value of less than 0.05 was considered significant. All data analysis was conducted using Prism Version 7 (GraphPad, La Jolla, CA, USA).

## 3. Results

### 3.1. Co-Culture Modifies bv2 Microglia and SH-sy5y Gene Expression and Ethanol-Induced Cytokines and Tlrs

**Figure 2:**
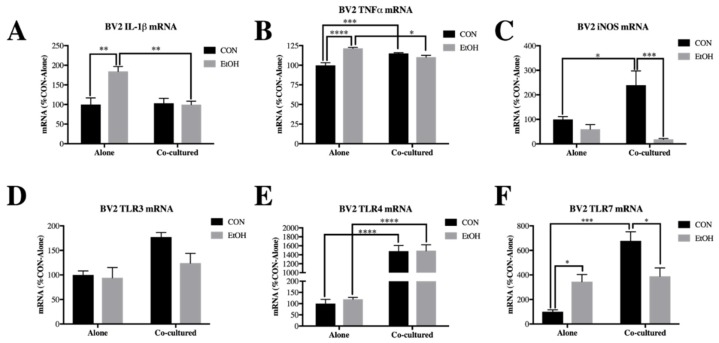
Co-culture alters ethanol-induced cytokines and Toll-like receptors (TLRs) in BV2 microglia. BV2 microglia were treated with ethanol (EtOH, 100 mM) for 24 h either alone or while co-cultured with SH-SY5Y. Cell lysates were examined for mRNA expression. (**A**) IL-1β expression was increased by EtOH in BV2 alone (185 ± 12%) but not in co-cultured BV2. (**B**) TNFα expression was increased by EtOH in BV2 alone (121 ± 1.2%) and co-culture (115 ± 0.9%) but not by EtOH in co-cultured BV2. (**C**) iNOS was increased by co-culture in BV2 (239 ± 58%), while EtOH significantly reduced iNOS in co-cultured cells (18 ± 4.0%). (**D**) TLR3 expression in BV2. **(E**) TLR4 expression was increased by co-culture (*p* < 0.0001). (**F**) TLR7 expression was increased by EtOH in BV2 alone (308 ± 59%), as well as by co-culture (678 ± 74%) but was significantly decreased by EtOH in co-cultured BV2 (389 ± 68%). Data is represented as %CON (control)-Alone ± SEM, *n* = 5–6 wells per group. * *p* < 0.05, ** *p* < 0.01, *** *p* < 0.001, **** *p* < 0.0001 vs. indicated group via Bonferroni’s post-hoc test following 2-way ANOVA.

**Figure 3:**
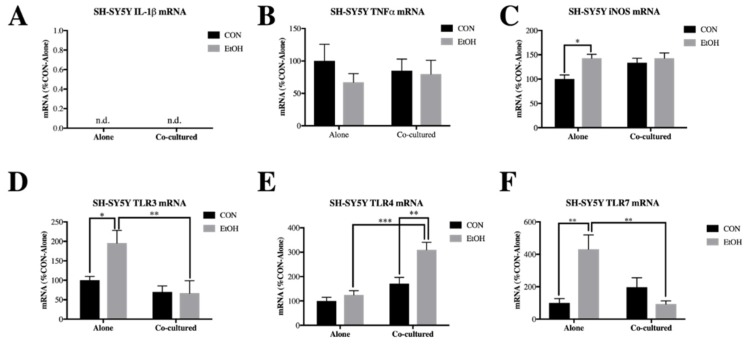
Co-culture alters ethanol-induced TLRs in SH-SY5Y. SH-SY5Y were treated with ethanol (EtOH, 100 mM) for 24 h alone or while co-cultured with BV2 microglia. Cell lysates were examined for mRNA expression. (**A**) IL-1β mRNA was not detected in SH-SY5Y cell lysates. (**B**) TNFα expression was not significantly affected by EtOH or co-culture in SH-SY5Y. (**C**) iNOS expression was significantly increased by EtOH in SH-SY5Y alone (143 ± 8.0%). (**D**) TLR3 expression was significantly increased by EtOH in SH-SY5Y alone (196 ± 32%) but not in co-cultured SH-SY5Y. (**E**) TLR4 expression was significantly increased by EtOH in co-cultured SH-SY5Y (310 ± 31%). (**F**) TLR7 was significantly increased by EtOH in SH-SY5Y alone (431 ± 89%) but not in co-cultured SH-SY5Y. Data is represented as %CON (control)-Alone ± SEM, *n* = 5–6 wells per group. n.d. = not detected (>40 cycles) * *p* < 0.05, ** *p* < 0.01, *** *p* < 0.001 vs. indicated group via Bonferroni’s post-hoc following 2-way ANOVA.

Co-cultures provide a model to understand glial-neuronal signaling via the release of soluble mediators [49]. We compared responses in BV2 microglia alone and SH-SY5Y neuroblastoma alone to co-cultured BV2/SH-SY5Y (see Figure 1 for co-culture schematic). Interestingly, co-culture of BV2 with SH-SY5Y caused significant changes in microglial gene expression (Figure 2, Table 2 and Supplementary Tables S1 and S2). Multiple neuroimmune receptors were increased in BV2 by co-culture with SH-SY5Y including TLR4 (15-fold, *p* < 0.0001), TLR7 (6.8-fold, *p* < 0.001), IL-13R (10-fold, *p* < 0.05), RAGE (3-fold, *p <* 0.0001) as well as statistically significant increases in iNOS, IL-4R, CXCR1, CD200R, Arg1, TNFα, TGFβ and multiple immune receptor intracellular signaling proteins (MD-2, TBK1, IKKβ). Among the genes measured in BV2 microglia, only the immunoreceptor DAP12 was decreased. In SH-SY5Y, co-culture with BV2 robustly increased several neuroimmune genes, such as RAGE (8.1-fold, *p* < 0.0001) and TGFβ (8.8-fold, *p* < 0.001), as well as neuronal markers such as doublecortin (DCX; 7.3-fold, *p* < 0.0001) and tyrosine hydroxylase (TH; 6.7-fold, *p* < 0.0001). Multiple other genes, including CXCL10, CD200, MCP-1, IL-13R, CD200, CX3CL1, DAP12 and HDAC2 were increased by co-culture in SH-SY5Y (Figure 3, Table 3 and Supplementary Tables S3 and S4). Of particular interest in these findings is the increase of microglial-neuronal signaling pathways, such as fractalkine (CX3CL1) and CD200, by co-culture alone. These findings suggest complex interactions of between these two cell types via the exchange of soluble mediators.

Previously, we found that ethanol treatment for 24 h upregulates cytokines IL-1β and TNFα, as well as TLR7 in BV2 microglia. In SH-SY5Y, ethanol did not affect cytokine expression but increased TLR3 and TLR7 expression [36]. However, we observed a significant main effect of co-culture (F [1,17] = 9.958, *p* < 0.01), ethanol (F [1,17] = 9.796, *p* < 0.01) and a co-culture x ethanol interaction (F [1,17] = 11.56, *p* < 0.01) on IL-1β expression in BV2 microglia (Supplementary Table S2). Post-hoc analysis indicated that in BV2 alone, ethanol increases expression of IL-1β (1.9-fold, *p* < 0.01), which was blocked when co-cultured with SH-SY5Y (Figure 2A). There was also a significant main effect of ethanol (F [1,17] = 11.56, *p* < 0.01) and a significant co-culture x ethanol interaction (F [1,19] = 34.6, *p* < 0.0001) on TNFα expression in BV2 microglia. Similar to IL1β, post-hoc analysis revealed that ethanol significantly elevated TNFα expression in BV2 alone (1.2-fold, *p* < 0.0001) but not in the BV2/SH-SY5Y co-cultures (Figure 2B). Similarly, pro-inflammatory mediator iNOS (main effect of ethanol, F [1,20] = 17.36, *p* < 0.001 and co-culture x ethanol interaction, F [1,20] = 8.366, *p* < 0.01) was unchanged by ethanol in BV2 microglia cultures alone but decreased in cells co-cultured with SH-SY5Y (Figure 2C). There was a significant main effect of co-culture on TLR3 (F [1,17] = 8.297, *p* < 0.05), TLR4 (F [1,20] = 227.6, *p* < 0.0001) and TLR7 (F [1,18] = 21.28, *p* < 0.001) in BV2 microglia. Furthermore, post-hoc analysis revealed that ethanol increased TLR7 expression in BV2 alone (3-fold, *p* < 0.05) which was blocked by co-culture (Figure 2D–F).

In SH-SY5Y, we found that even when co-cultured with BV2 microglia, ethanol had little effect on IL-1β and TNFα expression (Figure 3A,B). However, we did observe a small increase in iNOS expression by ethanol in SH-SY5Y alone (1.4-fold, *p* < 0.05), which was blocked by co-culture with the BV2 microglia (Figure 3C). There was a significant main effect of co-culture on TLR3 (F [1,16] = 15.92, *p* < 0.01) and TLR4 (F [1,18] = 29.74, *p* < 0.0001), significant main effect of ethanol on TLR3 (F [1,16] = 8.157, *p* < 0.05) and TLR4 (F [1,18] = 12, *p* < 0.01) and significant co-culture x ethanol interaction effect for TLR3 (F [1,16] = 5.431, *p* < 0.05), TLR4 (F [1,18] = 5.858, *p* < 0.05) and TLR7 (F [1,17] = 12.61, *p* < 0.01) in BV2 microglia. Interestingly, post-hoc analysis revealed that the increased expression of TLR3 (2-fold, *p* < 0.05) and TLR7 (4.3-fold, *p* < 0.01) by ethanol in SH-SY5Y alone was prevented in co-cultured cells, although TLR4 expression was actually increased by ethanol (3.1-fold, *p* < 0.01) in co-cultured SH-SY5Y (Figure 3D–F). We further examined a multitude of innate immune and other signaling genes in both BV2 microglia (Supplementary Tables S1 and S2) and SH-SY5Y (Supplementary Tables S3 and S4). These data indicate that co-culture between SH-SY5Y and BV2 alters ethanol-induced changes in innate immune gene expression.

**Figure 4:**
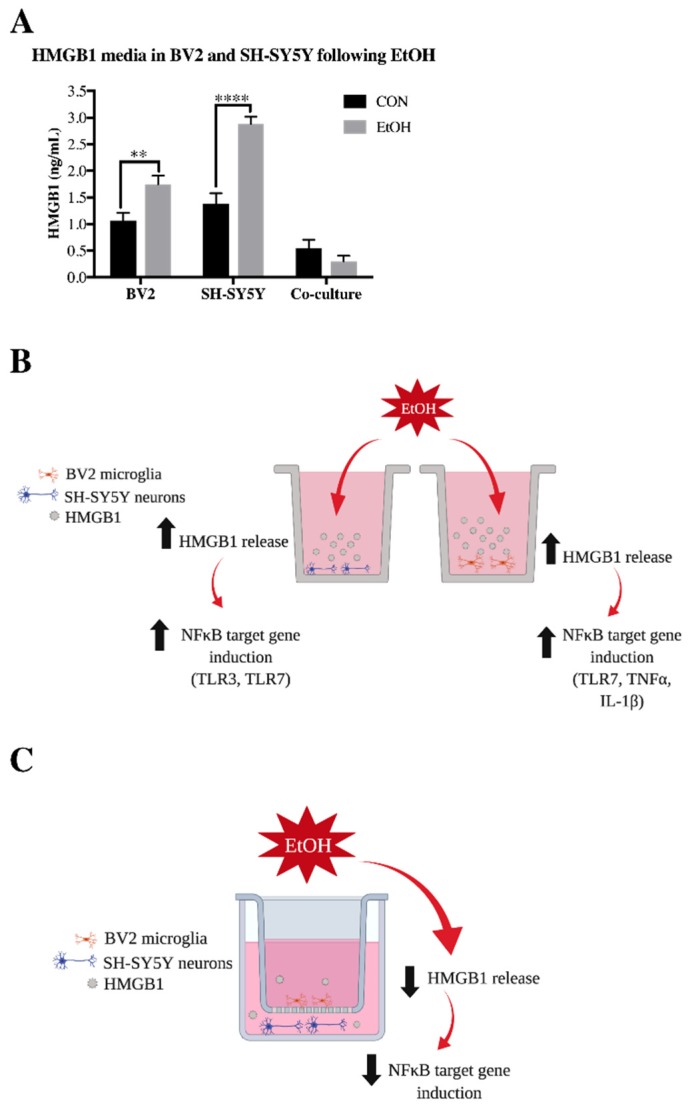
Co-culture blocks ethanol-induced HMGB1 release. BV2 microglia alone, SH-SY5Y alone and co-cultured BV2/SH-SY5Y were treated with ethanol (EtOH, 100 mM) for 24 h. Media was collected and analyzed for high mobility group box protein 1 (HMGB1) protein using enzyme-linked immunosorbent assay (ELISA). (**A**) HMGB1 was increased in the media of EtOH-treated BV2 microglia and SH-SY5Y but not in co-cultured cells. (**B**) Schematic diagraming EtOH increasing HMGB1 release in BV2 and SH-SY5Y alone, leading to increased expression of NFκB genes. (**C**) Schematic diagraming a lack of HMGB1 release in co-cultured BV2 and SH-SY5Y, preventing transcription of NFκB genes. *n* = 5–6 wells per group; ** *p* < 0.01, **** *p* < 0.0001. Schematic made using Biorender (biorender.com).

We previously found that HMGB1, an endogenous TLR agonist with cytokine-like activity when released, is released by ethanol treatment in both BV2 microglia and SH-SY5Y [36]. In order to investigate how co-culture alters this ethanol-induced release of HMGB1, we examined HMGB1 levels in the media of ethanol-treated BV2 microglia alone, SH-SY5Y alone, as well as BV2/SH-SY5Y co-cultures. We confirmed our previous findings of ethanol increasing HMGB1 release in both BV2 microglia and SH-SY5Y alone but interestingly, co-culture prevented ethanol-induced HMGB1 release (Figure 4A). As diagramed in Figure 4B,C, our findings indicate that while ethanol-induced HMGB1 release is correlated with an increase of multiple NFκB-regulated proinflammatory innate immune genes, co-culture blocks both HMGB1 and other proinflammatory genes.

### 3.2. Il-4 and IL-13 Expression Is Increased by Ethanol in Co-Cultured BV2 Microglia and SH-SY5Y

**Figure 5:**
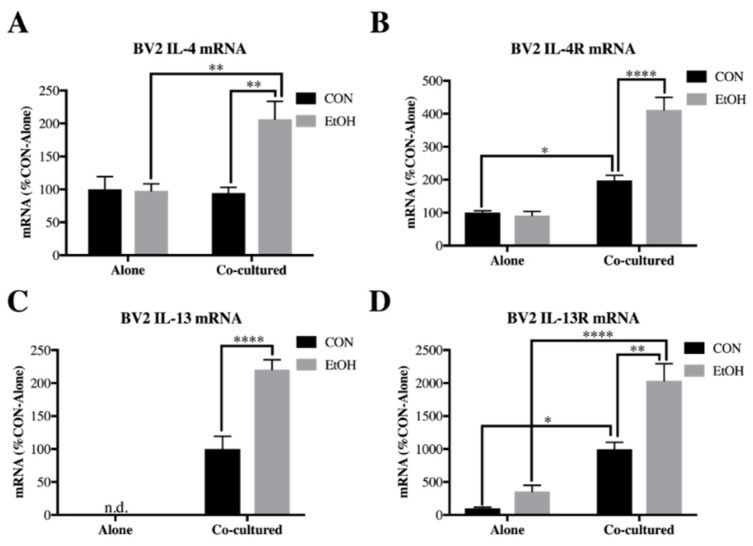
IL-4 and IL-13 is increased by ethanol in co-cultured BV2 microglia. BV2 microglia were treated with ethanol (EtOH, 100 mM) for 24 h either alone or while co-cultured with SH-SY5Y. Cell lysates were examined for mRNA expression. (**A**) IL-4 expression was increased by EtOH in co-cultured BV2 (207 ± 27%). (**B**) IL-4R expression was increased by co-culture (198 ± 15%), as well as by EtOH in co-cultured BV2 (412 ± 38%). (**C**) IL-13 was not detected in BV2 alone but was increased by EtOH in co-cultured BV2 (220 ± 15%). (**D**) IL-13R expression was increased by co-culture (997 ± 107%), as well as by EtOH in co-cultured BV2 (2034 ± 261%). Data is represented as %CON (control)-Alone ± SEM, *n* = 5–6 per group. n.d. = not detected (>40 cycles). * *p* < 0.05, ** *p* < 0.01, **** *p* < 0.0001 vs. indicated group via Bonferroni’s post-hoc following 2-way ANOVA.

In the periphery, cytokines IL-4 and IL-13 commonly modulate anti-inflammatory responses and share receptors that dimerize [53], resulting in complementary signaling pathways. While IL-4 and IL-13 have been discovered in brain [54] and have also been shown to affect dopaminergic neurons [21], little is known about this unconventional glial-neuronal signaling pathway. We examined both IL-4 and IL-13 ligands and receptors in our BV2 microglia/SH-SY5Y co-culture model to further study this pathway following ethanol treatment. In the BV2 microglia, both IL-4 and its receptor, IL-4R, had a main effect of co-culture (IL-4: F [1,18] = 6.966, *p* < 0.05; IL-4R: F [1,20] = 93.83, *p* < 0.0001), ethanol (IL-4: F [1,18] = 7.87, *p* < 0.05; IL-4R: F [1,20] = 22.62, *p* < 0.0001) and co-culture x ethanol interaction (IL-4: F [1,18] = 8.579, *p* < 0.01; IL-4R: F [1,20] = 26.6, *p* < 0.0001). Post-hoc analysis found that that ethanol only increased IL-4 (2-fold, *p* < 0.01) and IL-4 receptor (IL-4R; 2-fold, *p* < 0.0001) expression in cells co-cultured with SH-SY5Y (Figure 5A,B). Interestingly, IL-13 was not detectable in BV2 microglia alone but co-culture induced expression that was further increased by ethanol (2.2-fold, *p* < 0.0001, Figure 5C). The IL-13 receptor (IL-13R), had main effects of co-culture (F [1,17] = 55.98, *p* < 0.0001), ethanol (F [1,17] = 14.15, *p* < 0.01) and co-culture x ethanol interaction (F [1,17] = 5.141, *p* < 0.05) (Figure 5D).

**Figure 6:**
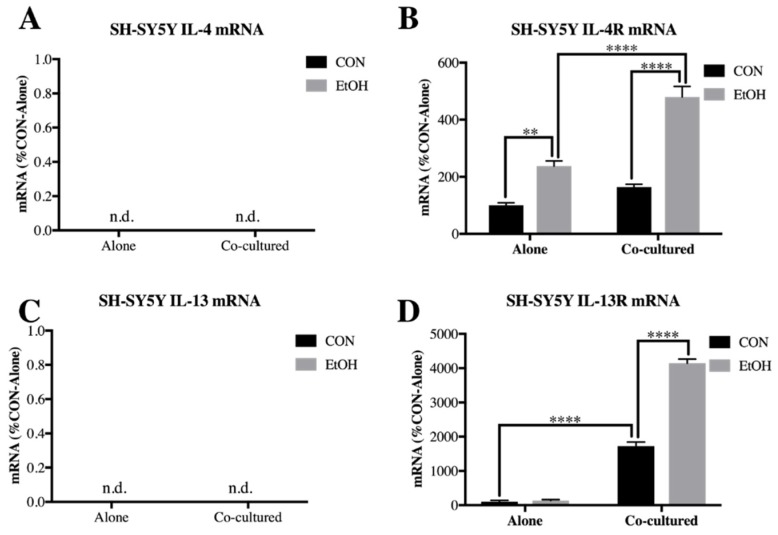
IL-4R and IL-13R is increased by ethanol in co-cultured *SH-SY5Y.* SH-SY5Y were treated with ethanol (EtOH, 100 mM) for 24 h either alone or while co-cultured with BV2 microglia. Cell lysates were examined for mRNA expression. (**A**) IL-4 expression was not detected in SH-SY5Y. (**B**) IL-4R was increased by EtOH in SH-SY5Y alone (238 ± 18%), as well as by EtOH in co-cultured SH-SY5Y (480 ± 37%). (**C**) IL-13 expression was not detected in SH-SY5Y. (**D**) IL-13R was increased in co-cultured SH-SY5Y (1723 ± 117%), as well as by EtOH in co-cultured SH-SY5Y (4147 ± 119%). Data is represented as %CON (control)-Alone ± SEM, *n* = 5–6 per group. n.d. = not detected (>40 cycles). ** *p* < 0.01, **** *p* < 0.0001 vs. indicated group via Bonferroni’s post-hoc following 2-way ANOVA.

In SH-SY5Y, mRNA for IL-4 and IL-13 were not detected within our detection threshold as opposed to BV2 (<40 cycles, Figure 6A,C). However, IL-4R had a main effect of co-culture (F [1,20] = 49.36, *p* < 0.0001), ethanol (F [1,20] = 108.3, *p* < 0.0001) and co-culture x ethanol interaction (F [1,20] = 16.7, *p* < 0.001). Post-hoc analysis indicated that IL-4R was increased by ethanol in SH-SY5Y alone (2.4-fold, *p* < 0.01), as well as further increased by ethanol in cells co-cultured with BV2 microglia (4.8-fold, *p* < 0.0001, Figure 6B). IL-13 receptor (IL-13R) similarly featured main effects of co-culture (F [1,13] = 563.9, *p* < 0.0001), ethanol (F [1,13] = 107.5, *p* < 0.0001) and co-culture interaction (F [1,13] = 101.2, *p* < 0.0001) and post-hoc analysis indicated IL-13R was increased by ethanol only in the co-culture setting in SH-SY5Y (41-fold, *p* < 0.0001, Figure 6D). These data indicate that IL-4 and IL-13 signaling is increased by ethanol in neuronal-microglial co-culture, suggesting a role for these cytokines in neuronal-glial ethanol-induced signaling.

### 3.3. IL-4 and IL-13 Reduce Ethanol-Induced Tnfα and IL-1β in Hippocampal-Entorhinal Slice Culture

**Figure 7:**
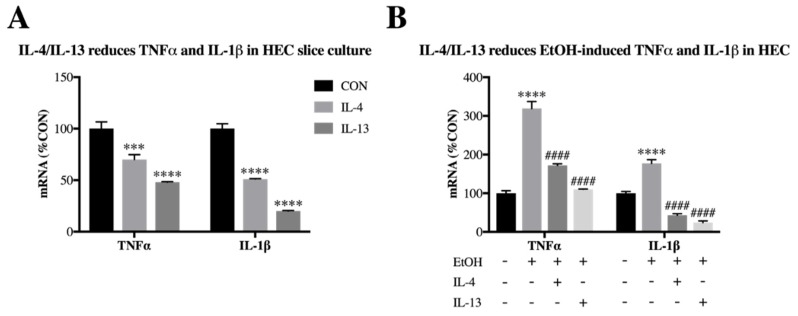
IL-4 and IL-13 reduce ethanol-induced TNFα and IL-1β in hippocampal entorhinal (HEC) slice culture. Hippocampal entorhinal (HEC) slice cultures were exposed to either IL-4 (500 ng/mL), IL-13 (1 ug/mL), and/or EtOH (100 mM) for 48 h. Tissue was processed for mRNA expression. (**A**) TNFα expression was reduced by IL-4 (70 ± 5%) and IL-13 (48 ± 1%). IL-1β expression was reduced by IL-4 (51 ± 1%) and IL-13 (20 ± 1%). (**B**) EtOH increased expression of TNFα (319 ± 18%), which was blocked by both IL-4 (172 ± 4%) and IL-13 (110 ± 1%). EtOH increased expression of IL-1β (177 ± 9.8%), which was blocked by both IL-4 (43 ± 4%) and IL-13 (24 ± 4%). Data is represented as %CON (control), *n* = 12 slices in each well per group. *** *p* < 0.001, **** *p* < 0.0001 vs. CON; ####*p* < 0.0001 vs. EtOH.

Previous experiments indicate that IL-4 reduces endotoxin-induced TNFα and IL-1β in human macrophages [55,56]; however, it is unclear how IL-4 and IL-13 may alter ethanol induction of these proinflammatory cytokines in brain. In order to investigate effects of IL-4 and IL-13 in an ex vivo setting containing all brain cell types, we treated hippocampal-entorhinal (HEC) brain slices from postnatal day 7 rats with IL-4 and IL-13 recombinant proteins. Both IL-4 and IL-13 reduced TNFα (0.7-fold, *p* < 0.001 and 0.48-fold, *p* < 0.0001, respectively) as well as IL-1β (0.51-fold, *p* < 0.0001 and 0.2-fold, *p* < 0.0001, respectively) mRNA expression (Figure 7A). We next treated the HEC slices with EtOH (100 mM, 48 h) either with or without IL-4 and IL-13 proteins. We found that while EtOH increased expression of both TNFα (3.2-fold, *p* < 0.0001) and IL-1β (1.8-fold, *p* < 0.0001), both IL-4 and IL-13 reduced this increase (Figure 7B). These data indicate that IL-4 and IL-13 are capable of reducing ethanol-induced proinflammatory cytokines TNFα and IL-1β in brain.

### 3.4. IL-10 and Tgfβ Are Increased by Ethanol in Co-Cultured BV2 Microglia and SH-SY5Y

**Figure 8:**
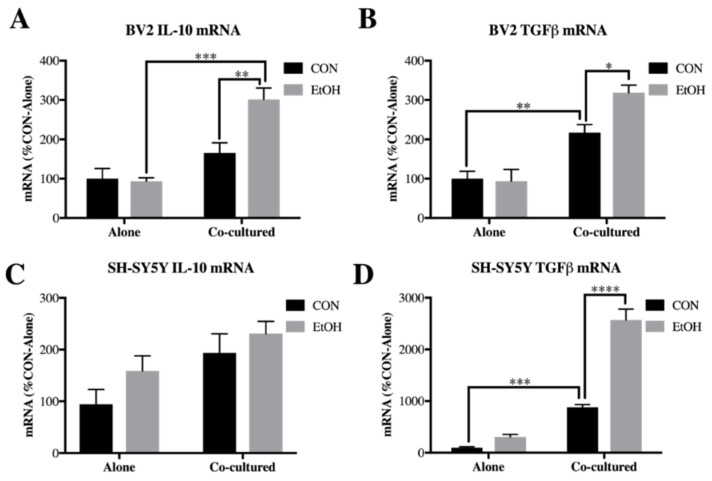
IL-10 and TGFβ are increased by ethanol in co-cultured BV2 microglia and SH-SY5Y. BV2 microglia and SH-SY5Y were treated with ethanol (EtOH, 100 mM) for 24 h either alone or while co-cultured. Cell lysates were examined for mRNA expression. (**A**) IL-10 expression was increased by EtOH in co-cultured BV2 (182 ± 17%). (**B**) TGFβ was increased by co-culture (217 ± 20%) and by EtOH in co-cultured BV2 (319 ± 19%). (**C**) IL-10 expression in SH-SY5Y. (**D**) TGFβ expression was increased by co-culture (880 ± 53%) and by EtOH in co-cultured SH-SY5Y (2571 ± 221%). Data is represented as %CON (control)-Alone, *n* = 5–6 per group. * *p* < 0.05, ** *p* < 0.01, *** *p* < 0.001, **** *p* < 0.0001 via Bonferroni’s post-hoc following 2-way ANOVA.

IL-10 and TGFβ are both cytokines that are activated downstream of IL-4 and IL-13 signaling [26,57,58] and play roles in learning, memory and neuroprotection [31,34] that may be impacted by alcohol pathology. To determine whether these genes are impacted by neuronal-glial ethanol induced signaling, we examined expression of IL-10 and TGFβ in our co-culture model. In BV2 microglia, both IL-10 and TGFβ had significant main effects of co-culture (IL-10: F [1,18] = 27.28, *p* < 0.0001; TGFβ: F [1,20] = 57.65, *p* < 0.0001), ethanol (IL-10: F [1,18] = 6.116, *p* < 0.05; TGFβ: F [1,20] = 4.459, *p* < 0.05) and co-culture x ethanol interaction (IL-10: F [1,18] = 7.441, *p* < 0.05; TGFβ: F [1,20] = 5.726). Post-hoc analysis revealed that IL-10 (1.8-fold, *p* < 0.01) and TGFβ (3.2-fold, *p* < 0.05) were increased by ethanol only when co-cultured with SH-SY5Y (Figure 8A,B). A main effect of co-culture on IL-10 expression was found in SH-SY5Y (F [1,17] = 7.692, *p* < 0.05), as well as a significant main effect of co-culture (F [1,20] = 185.3, *p* < 0.0001), ethanol (F [1,20] = 72.22, *p* < 0.0001) and co-culture x ethanol interaction (F [1,20] = 43.97, *p* < 0.0001) for TGFβ. Post-hoc analysis revealed that ethanol significantly induced TGFβ only in the co-cultured SH-SY5Y (Figure 8C,D). Overall, these data indicate that genes downstream of IL-4 and IL-13 signaling, TGFβ and IL-10, are induced in neuronal-glial cultures, further indicating a role for the IL-4 and IL-13 pathway induction in these cells.

## 4. Discussion

**Figure 9:**
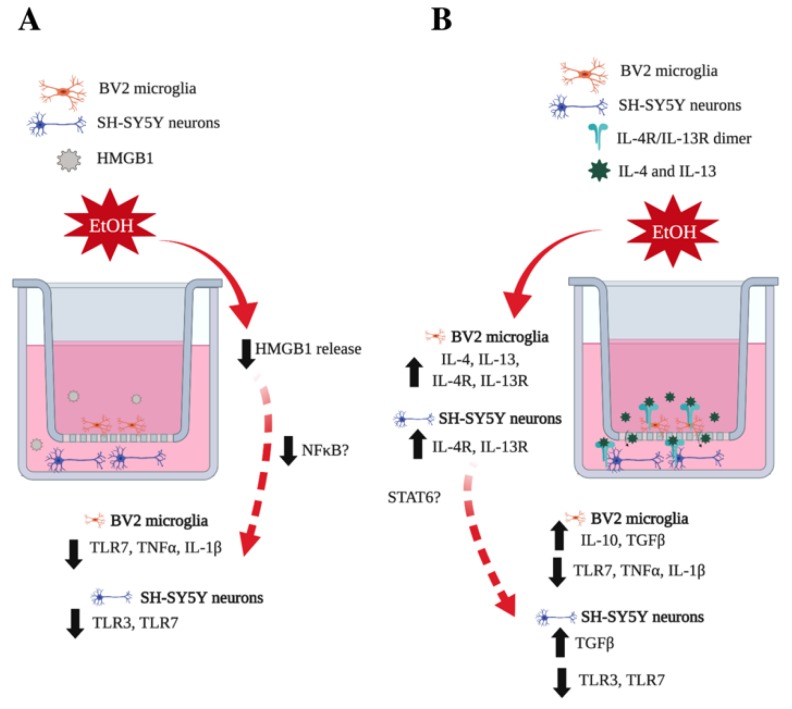
Ethanol increases IL-4 and IL-13 signaling between microglia and neurons. (**A**) Schematic illustrating a lack of HMGB1 release in co-cultured neurons and microglia that is correlated with a decrease in pro-inflammatory cytokine and receptors, possibly via altered NFκB activity. (**B**) Schematic illustrating ethanol-induced IL-4 and IL-13 signaling between BV2 microglia and SH-SY5Y. Ethanol increases IL-4 and IL-13 in BV2 microglia, as well as IL-4R and IL-13R in SH-SY5Y. Downstream targets of IL-4 and IL-13, such as IL-10 and TGFβ, are also increased in co-cultured cells and pro-inflammatory cytokines and receptors are decreased, possibly via a STAT6-mediated mechanism. Schematic made using Biorender (biorender.com).

This study demonstrates for the first time that co-culture of SH-SY5Y and BV2 alters ethanol-induced expression of innate immune signaling molecules. Co-culture with SH-SY5Y prevents ethanol induction of cytokines TNFα and IL-1β in BV2 microglia. Similarly, co-culture with BV2 microglia prevents ethanol-induced increases in TLR3 and TLR7 in SH-SY5Y. This was accompanied by a lack of ethanol induction of HMGB1 in the media of the co-cultured cells. Previous studies have indicated that HMGB1 facilitates the activation of multiple TLRs, including TLR3, TLR4 and TLR7 [5,7]. Therefore, the lack of HMGB1 induction in co-cultured BV2 microglia and SH-SY5Y may play a role in the lack of TLR and NFκB induced genes, including TNFα and IL-1β (see diagram in Figure 9A). Furthermore, we found that co-culture in conjunction with ethanol increased several known microglial-neuronal signaling pathways, such as fractalkine and CD200. Fractalkine (CX3CL1) attenuates lipopolysaccharide-induced increases in TNFα in rat primary microglia [59], suggesting that BV2 microglial CX3CR1 activation may underlie some of the effects seen in our co-culture model. Similarly, in a Aβ model of inflammation, addition of neurons into microglial cultures inhibited induction of IL-1β and TNFα, which was blocked with a CD200 blocking antibody [60]. These studies suggest that future research should examine the possible contribution of fractalkine and CD200 in ethanol-induced innate immune signaling between cell types. 

Our findings were surprising in that previous studies indicate ethanol induces HMGB1 as well as various pro-inflammatory cytokines and TLRs in SH-SY5Y cultures alone, hippocampal entorhinal brain slice culture and in brain [13,19,20]. Importantly, while most of our findings consist of mRNA data, our previous studies have indicated a correspondence between mRNA and protein for targets such as TNFα, IL-1 β and TLR7 [14,20]. However, a similar inhibition of innate immune activation in SH-SY5Y due to lipopolysaccharide + interferons or Aβ has been observed when co-cultured with THP-1 monocytes [61]. Our results therefore suggest a unique and specific mechanism of inhibiting HMGB1 and other TLR/cytokine expression that exists as a paracrine signaling mechanism between SH-SY5Y and BV2 microglia. Astrocytes, as well as direct cell-to-cell contact, may modulate this response in brain, leading to increased HMGB1 and pro-inflammatory cytokines observed in slice culture and *in vivo*. Indeed, certain microglial-neuronal signaling molecules, such as fractalkine, are neuroprotective when in soluble form whereas membrane forms are pro-inflammatory [62]. However, further research is needed to explore the unique modulatory effects of neuronal-microglial co-cultures and whether our findings are found in primary neuronal and microglial cultures.

IL-4 and IL-13 are both described as anti-inflammatory cytokines in the periphery and in microglia these cytokines have been found to promote an M2 phenotype [63,64]. However, the impact of ethanol on IL-4 and IL-13 signaling has been poorly understood. We observed for the first time that ethanol induced both IL-4 and IL-13 and their receptors on BV2 microglia only when in the presence of SH-SY5Y. Interestingly, we also observed that IL-13 was not detectable in BV2 alone, paralleling previous studies that found that endotoxin induces IL-13 in primary microglia co-cultured with neurons but not alone or with astrocytes [27]. Interesting new research also finds that IL-4 and IL-13 may play roles in learning and memory [29,30], suggesting a role for neurons in IL-4 and IL-13 signaling. Furthermore, a novel role for neuronal IL-13Rα1 was recently discovered, in which stimulation of this receptor led to dopaminergic cell loss [28]. In our study, we confirmed that SH-SY5Y IL-4 and IL-13 signaling is enhanced following ethanol treatment in BV2/SH-SY5Y microglial co-cultures, with an increased induction of IL-4R and IL-13R by ethanol in co-cultured SH-SY5Y. Our data indicates that interactions between the two cell types induces IL-4 and IL-13 signaling in response to ethanol, a finding which may have relevance in both neuroprotection and learning, although further studies are needed to translate these findings *in vivo*. Our findings may represent a specific feature of microglial-neuronal signaling that may occur via the secretion of soluble mediators.

IL-4 and IL-13 both reduce endotoxin-induced IL-1β and TNFα in peripheral macrophages [55,56], suggesting that in brain these cytokines may inhibit the expression of pro-inflammatory cytokines. Indeed, we confirmed using hippocampal-entorhinal slice culture that both IL-4 and IL-13 not only decrease expression of IL-1β and TNFα but also inhibit ethanol induction of these cytokines. In brain, excessive expression of IL-1β and TNFα has been linked to disruption of long-term potentiation [65,66], an important mechanism that regulates learning and memory formation. Pro-inflammatory cytokines are also linked to neurodegeneration [67]. Therefore, induction of IL-4 and IL-13 signaling may underlie the lack of induction of pro-inflammatory cytokines in BV2/SH-SY5Y microglial co-culture.

We also found that downstream mediators of IL-4 and IL-13 signaling, TGFβ and IL-10, are induced by ethanol in co-cultured BV2 microglia, with TGFβ being induced in co-cultured SH-SY5Y. Although the exact mechanism of this activity is unknown, IL-4 and IL-13 signaling in peripheral macrophages activates transcription factor STAT6 [68]. IL-4 has also been shown to activate STAT6 in BV2 microglia [69]. This suggests that IL-4 and IL-13 may be signaling in a STAT6 dependent manner to promote expression of TGFβ and IL-10 (see Figure 9B); however further studies are needed to elucidate these responses.

As stated above, it is important to note that this study uses immortalized cell lines for the co-culture experiments. SH-SY5Y is a well-characterized neuronal line used for modeling dopaminergic neurons in Parkinson’s disease [70] and BV2 are commonly used to model microglial immune responses, exhibiting a similar response as primary microglia upon lipopolysaccharide stimulation [71]. However, these findings should be investigated in primary neuronal and microglial co-cultures. Our co-culture model provides insight into signals that might occur in vivo via the release of soluble mediators but lacks cell-cell contact and the presence of other cell types. Our findings in rat primary hippocampal-entorhinal slice culture provides further evidence for the anti-inflammatory role of IL-4 and IL-13 in brain. Whether this signaling system is induced by ethanol in vivo should be explored. 

## 5. Conclusions

Overall, our findings indicate a role for IL-4 and IL-13 in ethanol-induced signaling between BV2 microglia and SH-SY5Y. Interestingly, we also observed an overall effect of co-culture on BV2 microglia and SH-SY5Y gene expression, indicating that signaling molecules between the two cell types even in basal settings influence their activity. Further research is needed to elucidate these signaling mechanisms. We further show that ethanol induction of TLR agonist HMGB1 is blocked by co-cultured neurons and microglia. These data reveal that neuronal-microglial signaling is altered by ethanol and indicate that more research is needed to further explore these cell-to-cell signaling mechanisms and how they play a role in alcohol pathology.

## Figures and Tables

**Table 1:** Primers used for real-time polymerase chain reaction.

Gene	Species	Forward (5′–3′)	Reverse (5′–3′)
ADAM10	Human	ATGGGAGGTCAGTATGGGAATC	ACTGCTCTTTTGGCACGCT
β-Actin	Human	GAT GCA GAA GGA GAT CAC TGC	ATA CTC CTG CTT GCT GAT CCA
Caspase 3	Human	CTCTGGTTTTCGGTGGGTGT	GCTGCATCGACATCTGTACC
CCR2	Human	CCACATCTCGTTCTCGGTTTATC	CAGGGAGCACCGTAATCATAATC
CD200	Human	ACGTCTGTTACCAGCATCCTC	CTTAAAGTCGGTCACAGTCCC
ChAT	Human	GGA GGT GGA GGG TTT GTG AC	ATT TCC TTG GCA CCC TGA GG
CX3CL1	Human	ACCACGGTGTGACGAAATG	TGTTGATAGTGGATGAGCAAAGC
CXCL10	Human	GACTCTGAGTGGAACTCAAGGAAT	GTGGCAATGATCTCAACACG
DAP12	Human	ACTGAGACCGAGTCGCCTTAT	ATACGGCCTCTGTGTGTTGAG
DCX	Human	TTCAAGGGGATTGTGTACGCT	GTCAGACAGAGATCGCGTCAG
DR3	Human	AGAGATACTGACTGTGGGACC	CCCAGAACACACCTACTCTGC
FADD	Human	GAAAACGCGCTCTTGTCGAT	GCCCGAGGCATAGGAACTTG
Fas	Human	GTCTCCTGCGATGTTTGGC	TTCAAGGAAAGCTGATACCTATTTC
HDAC1	Human	CCGCATGACTCATAATTTGCTG	ATTGGCTTTGTGAGGGCGATA
HDAC2	Human	ATGGCGTACAGTCAAGGAGG	TGCGGATTCTATGAGGCTTCA
HMGB1	Human	GGA GAT CCT AAG AAG CCG AGA	CAT GGT CTT CCA CCT CTC TGA
HMOX1	Human	AAGACTGCGTTCCTGCTCAAC	AAAGCCCTACAGCAACTGTCG
IKKβ	Human	CTGGCCTTTGAGTGCATCAC	CGCTAACAACAATGTCCACCT
IL-10	Human	GACTTTAAGGGTTACCTGGGTTG	TCACATGCGCCTTGATGTCTG
IL-13	Human	GAGGATGCTGAGCGGATTCTG	CACCTCGATTTTGGTGTCTCG
IL-13R	Human	ACAACCTGAGCTACATGAAGTG	GGCTTCTGTGCCAATAGTAGAG
IL-1β	Human	ATG ATG GCT TAT TAC AGT GGC AA	GTCGGAGATTCGTAGCTGGA
IL-4	Human	CCAACTGCTTCCCCCTCTG	TCTGTTACGGTCAACTCGGTG
IL-4R	Human	ACACCAATGTCTCCGACACTC	TGTTGACTGCATAGGTGAGATGA
iNOS	Human	TTCAGTATCACAACCTCAGCAAG	TGGACCTGCAAGTTAAAATCCC
MCP1	Human	CTCTCGCCTCCAGCATGAAA	AGGGTGTCTGGGGAAAGCTA
MD-2	Human	GAA GCT CAG AAG CAG TAT TGG GTC	GGT TGG TGT AGG ATG ACA AAC TCC
NGFR	Human	CCTACGGCTACTACCAGGATG	CACACGGTGTTCTGCTTGT
RAGE	Human	CTA CCG AGT CCG TGT CTA CCA	CAT CCA AGT GCC AGC TAA GAG
TBK1	Human	TGGGTGGAATGAATCATCTACGA	GCTGCACCAAAATCTGTGAGT
TGFβ	Human	CAATTCCTGGCGATACCTCAG	GCACAACTCCGGTGACATCAA
TH	Human	GGAAGGCCGTGCTAAACCT	GGATTTTGGCTTCAAACGTCTC
TL1A	Human	TGCAGGACTCACCACATA	CTTGGCTTATCTCCGTCT
TLR3	Human	TTGCCTTGTATCTACTTTTGGGG	TCAACACTGTTATGTTTGTGGGT
TLR4	Human	CCTGCGTGGAGGTGGTTCC	AGAGGTGGCTTAGGCTCTGATA
TLR7	Human	GATAACAATGTCACAGCCGTCC	GTTCCTGGAGTTTGTTGATGTTC
TNFα	Human	CCC AGG CAG TCA GAT CAT CTT CT	ATG AGG TAC AGG CCC TCT GAT
TREM1	Human	GAACTCCGAGCTGCAACTAAA	TCTAGCGTGTAGTCACATTTCAC
TREM2	Human	GGTCAGCACGCACAACTTG	CGCAGCGTAATGGTGAGAGT
Arg1	Mouse	TTAGGCCAAGGTGCTTGCTGCC	TACCATGGCCCTGAGGAGGTTC
β-Actin	Mouse	GTA TGA CTC CAC TCA CGG CAA A	GGT CTC GCT CCT GGA AGA TG
CCR2	Mouse	ATCCACGGCATACTATCAACATC	CAAGGCTCACCATCATCGTAG
CD200R	Mouse	TAAGGTGGAGGCATTTCCAGT	GATTCCAATGGCCGACAAAGTA
CX3CR1	Mouse	TCTTCACGTTCGGTCTGGTG	TGCACTGTCCGGTTGTTCAT
DAP12	Mouse	GAGTGACACTTTCCCAAGATGC	CCTTGACCTCGGGAGACCA
DR3	Mouse	CTCACCTTTCTCTTGTGTCCC	GTGCAGTCATTGCCACGTAT
HMGB1	Mouse	CGC GGA GGA AAA TCA ACT AA	TCA TAA CGA GCC TTG TCA GC
IKKβ	Mouse	GGAGCCTGGGAAATGAAAGAA	GCCAGAGCCCTACCTGATTG
IL-10	Mouse	GGC AGA GAA GCA TGG CCC AGA A	GGC TGA GGC GCT GTC ATC GAT T
IL-13	Mouse	CCTGGCTCTTGCTTGCCTT	GGTCTTGTGTGATGTTGCTCA
IL-13R	Mouse	TCAGCCACCTGTGACGAATTT	TGAGAGTGCAATTTGGACTGG
IL-1β	Mouse	CTG GTG TGT GAC GTT CCC ATT A	CCG ACA GCA CGA GGC TTT
IL-4	Mouse	TGGGTCTCAACCCCCAGCTAGT	TGCATGGCGTCCCTTCTCCTGT
IL-4R	Mouse	TCTGCATCCCGTTGTTTTGC	GCACCTGTGCATCCTGAATG
iNOS	Mouse	GTGGTGACAAGCACATTTGG	CATGGTGAACACGTTCTTGG
MCP1	Mouse	ACT GAA GCC AGC TCT CTC TTC CTC	TTC CTT CTT GGG GTC AGC ACA GAC
MD-2	Mouse	CGC TGC TTT CTC CCA TAT TGA	CCT CAG TCT TAT GCA GGG TTC A
RAGE	Mouse	GAA GGC TCT GTG GGT GAG TC	CCG CTT CCT CTG ACT GAT TC
TBK1	Mouse	ACTGGTGATCTCTATGCTGTCA	TTCTGGAAGTCCATACGCATTG
TGFβ	Mouse	CTCCCGTGGCTTCTAGTGC	GCCTTAGTTTGGACAGGATCTG
TLR3	Mouse	GTG AGA TAC AAC GTA GCT GAC TG	TCC TGC ATC CAA GAT AGC AAG T
TLR4	Mouse	ATG GCA TGG CTT ACA CCA CC	GAG GCC AAT TTT GTC TCC ACA
TLR7	Mouse	ATG TGG ACA CGG AAG AGA CAA	GGT AAG GGT AAG ATT GGT GGT G
TNFα	Mouse	GAC CCT CAC ACT CAG ATC ATC TTC T	CCT CCA CTT GGT GGT TTG CT
TREM1	Mouse	GACTGCTGTGCGTGTTCTTTG	GCCAAGCCTTCTGGCTGTT
TREM2	Mouse	CTGGAACCGTCACCATCACTC	CGAAACTCGATGACTCCTCGG
β-Actin	Rat	CTACAATGAGCTGCGTGT	CAGGTCCAGACGCAGGAT
IL-1β	Rat	TTGTGCAAGTGTCTGAAGCA	TGTCAGCCTCAAAGAACAGG
TNFα	Rat	ATGTGGAACTGGCAGAGGAG	ACGAGCAGGAATGAGAAGAGG

**Table 2:** Summary of effects of ethanol and co-culture on gene expression in BV2 microglia.

BV2 Microglia				
Gene	CON-Alone	EtOH-Alone	CON-CC	EtOH-CC
NFκB signaling genes				
CCR2	100 ± 11	185 ± 29 *	177 ± 23 ns	294 ± 16@@, ##
HMGB1	100 ± 12	109 ± 13	107 ± 6.9	67 ± 8.3
IKKβ	100 ± 29	90 ± 21	367 ± 11 ****	500 ± 45@@@@,#
MCP1	100 ± 15	62 ± 21	58 ± 5.6	19 ± 0.7#
MD-2	100 ± 21	147 ± 22 ns	187 ± 5.9 *	173 ± 22
RAGE	100 ± 8.5	117 ± 12	342 ± 55 ***	340 ± 33@@@
TBK1	100 ± 23	106 ± 22	631 ± 61 ****	666 ± 65@@@@
Microglial markers				
Arg1	100 ± 21	73 ± 15	160 ± 5.6 *	137 ± 5.3@
CD200R	100 ± 27	217 ± 46 ns	320 ± 27 *	658 ± 71@@@@,###
CX3CR1	100 ± 23	122 ± 20	203 ± 32 *	234 ± 20@
TREM signaling genes				
DAP12	100 ± 6.6	187 ± 8.2 ****	75 ± 2.3 **	124 ± 4.4@@@@, ####
TREM1	100 ± 11	114 ± 18	401 ± 34 ns	1144 ± 199@@@, ##
TREM2	100 ± 13	138 ± 3	86 ± 8.9	1 25 ± 7.1
TNF superfamily genes				
DR3	100 ± 12	199 ± 36 ns	341 ± 24 ns	2261 ± 429@@@@, ####

(red) * *p* < 0.05, ** *p* < 0.1, *** *p* < 0.001, **** *p* < 0.0001 vs. CON-Alone; (blue) @*p* < 0.05, @@*p* < 0.01, @@@*p* <0.001, @@@@*p* < 0.0001 vs. EtOH-Alone; (green) #*p* < 0.05, ##*p* < 0.01, ###*p* < 0.001, ####*p* < 0.0001 vs. CON-CC. CON = control, EtOH = ethanol, CC = co-culture, ns-not statistically significant. Data was analyzed via two-way ANOVA followed by post-hoc Bonferroni’s test (see Supplementary Tables S1 and S2 for complete statistics).

**Table 3:** Summary of effects of ethanol and co-culture on gene expression in SH-SY5Y.

SH-SY5Y neuroblastoma				
Gene	CON-Alone	EtOH-Alone	CON-CC	EtOH-CC
NFκB signaling genes				
CCR2	100 ± 24	187 ± 26	98 ± 14	142 ± 45
CXCL10	100 ± 32	84 ± 12	210 ± 32 *	85 ± 18#
HMGB1	100 ± 5.9	71 ± 8.9	42 ± 12	102 ± 22#
HMOX1	100 ± 8.5	580 ± 95 ****	126 ± 7.5	186 ± 25 ns
IKKβ	100 ± 7.5	258 ± 57 *	202 ± 12	490 ± 47@@, ###
MCP1	100 ± 29	42 ± 11	180 ± 19 *	77 ± 7.7##
MD-2	n.d.	n.d.	n.d.	n.d.
RAGE	100 ± 6.2	233 ± 38 ns	810 ± 63 ****	796 ± 114@@@
TBK1	100 ± 33	185 ± 47	243 ± 24 ns	642 ± 107@@@,##
TNF superfamily and death receptor pathway genes				
Caspase 3	100 ± 19	243 ± 34 ns	525 ± 78 **	636 ± 134@@
DR3	100 ± 33	178 ± 33	561 ± 28 ****	702 ± 70@@@@
FADD	100 ± 7.1	124 ± 11.5	171 ± 18	330 ± 58@@@, #
Fas	100 ± 11.8	861 ± 352 ns	1203 ± 357 ns	2422 ± 238@
TL1A	n.d.	n.d.	100 ± 19	43 ± 7.1#
NGFR	100 ± 12	5127 ± 471****	42 ± 1.7	331 ± 80@@@@
Neuron-specific genes				
CD200	100 ± 26	183 ± 61	290 ± 14 **	604 ± 67@@@@,###
CX3CL1	100 ± 6.3	123 ± 7.0	213 ± 17 ****	274 ± 12@@@@, ##
ChAT	100 ± 16	144 ± 43	177 ± 29	234 ± 38
DCX	100 ± 26	108 ± 32	729 ± 40 ****	732 ± 54@@@@
TH	100 ± 15	124 ± 40	668 ± 113 ****	110 ± 27####
TREM signaling genes				
DAP12	100 ± 20	70 ± 5.2	13701 ± 3140 ***	11800 ± 1552@@@
TREM1	100 ± 23	177 ± 45	485 ± 221	1506 ± 343@@@, #
TREM2	100 ± 23	140 ± 40	100 ± 14	172 ± 25
Proteases				
ADAM10	100 ± 33	199 ± 65	700 ± 56 **	1429 ± 161@@@@, ###
Histone deacetylases				
HDAC1	100 ± 11.7	131 ± 7.0	185 ± 8.8	336 ± 64@@
HDAC2	100 ± 11.7	193 ± 23	354 ± 72 *	378 ± 84

(red) * *p* < 0.05, ** *p* < 0.1, *** *p* < 0.001, **** *p* < 0.0001 vs. CON-Alone; (blue) @*p* < 0.05, @@*p* < 0.01, @@@*p* < 0.001, @@@@*p* < 0.0001 vs. EtOH-Alone; (green) #*p* < 0.05, ##*p* < 0.01, ###*p* < 0.001, ####*p* < 0.0001 vs. CON-CC. CON = control, EtOH = ethanol, CC = co-culture. n.d. = not detectable (>40 cycles). Data was analyzed via two-way ANOVA followed by post-hoc Bonferroni’s test.

## Supplementary Materials

The following are available online at https://www.mdpi.com/2076-3425/9/9/228/s1; Table S1. Post-hoc Bonferroni analyses of BV2 microglial genes +/− co-culture with SH-SY5Y neuroblastoma; Table S2. Table of statistics (F-values, *p*-values) for BV2 co-culture data; Table S3. Post-hoc Bonferroni analyses of SH-SY5Y neuroblastoma +/− BV2 microglia co-culture; Table S4. Table of statistics (F-values, *p*-values) for SH-SY5Y neuroblastoma co-culture data.
