# Supplementary material for: Ethanol Induction of Innate Immune Signals Across BV2 Microglia and SH-SY5Y Neuroblastoma Involves Induction of IL-4 and IL-13
Source: Brain Sci. 2019 Sep 10;9(9):228. doi: 10.3390/brainsci9090228 (PMC6770440; doi:10.3390/brainsci9090228)
Supplement: Supplementary file 1 [file brainsci-09-00228-s001.zip › brainsci-590085-supplementary.docx]

**Supplementary Table S1.** Post-hoc Bonferroni analyses of BV2 microglial genes +/- co-culture with SH-SY5Y neuroblastoma.

| **Gene** | **Alone CON**  **vs.**  **Alone EtOH**  **P-Value (Bonferroni)** | **Alone CON**  **vs.**  **Co-Cultured CON**  **P-value (Bonferroni)** | **Alone EtOH**  **vs.**  **Co-Cultured EtOH**  **P-Value (Bonferroni)** | **Co-Cultured CON**  **vs.**  **Co-Cultured EtOH**  **P-Value (Bonferroni)** |
| --- | --- | --- | --- | --- |
| Arg1 | >0.9999 | 0.043* | 0.0441* | >0.9999 |
| CCR2 | 0.0493 | 0.1146 | 0.0112* | 0.0091** |
| CD200R | 0.5469 | 0.0196* | <0.0001**** | 0.0003*** |
| CX3CR1 | >0.9999 | 0.0393* | 0.0228* | >0.9999 |
| DAP12 | <0.0001**** | 0.0086** | <0.0001**** | <0.0001**** |
| DR3 | >0.9999 | >0.9999 | <0.0001**** | <0.0001**** |
| HMGB1 | >0.9999 | >0.9999 | 0.0583 | 0.0749 |
| IKKβ | >0.9999 | <0.0001**** | <0.0001**** | 0.0261* |
| IL-10 | >0.9999 | 0.4657 | 0.0003*** | 0.0063** |
| IL-13R | >0.9999 | 0.014* | <0.0001**** | 0.0015** |
| IL-1β | 0.0029** | >0.9999 | 0.0019** | >0.9999 |
| IL-4 | >0.9999 | >0.9999 | 0.0091** | 0.0026** |
| IL-4R | >0.9999 | 0.0268* | <0.0001**** | <0.0001**** |
| iNOS | >0.9999 | 0.0304* | >0.9999 | 0.0004*** |
| MCP1 | 0.3497 | 0.212 | 0.1934 | 0.3203 |
| MD-2 | 0.6428 | 0.0159* | >0.9999 | >0.9999 |
| RAGE | >0.9999 | 0.0002*** | 0.0006*** | >0.9999 |
| TBK1 | >0.9999 | <0.0001**** | <0.0001**** | >0.9999 |
| TGFβ | >0.9999 | 0.009** | <0.0001**** | 0.0279* |
| TLR3 | >0.9999 | 0.0949 | >0.9999 | 0.2236 |
| TLR4 | >0.9999 | <0.0001**** | <0.0001**** | >0.9999 |
| TLR7 | 0.0277* | 0.0001*** | >0.9999 | 0.0226* |
| TNFα | <0.0001**** | 0.0006*** | 0.0166* | 0.7509 |
| TREM1 | >0.9999 | 0.6695 | 0.0002*** | 0.0017** |
| TREM2 | 0.0874 | >0.9999 | >0.9999 | 0.0537 |

**Supplementary Table S2.** Table of statistics (F-values, *p*-values) for BV2 co-culture data.

|  | **F-Values** | | | **P-Values** | | |
| --- | --- | --- | --- | --- | --- | --- |
| **Gene** | **Interaction (Co-Culture × Ethanol)** | **Effect of Co-Culture** | **Effect of Ethanol** | **Interaction (Co-Culture × Ethanol)** | **Effect of Co-Culture** | **Effect of Ethanol** |
| Arg1 | F (1, 17) = 0.01674 | F (1, 17) = 18.57 | F (1, 17) = 3.044 | P = 0.8986 | P = 0.0005 | P = 0.0991 |
| CCR2 | F (1, 18) = 0.5699 | F (1, 18) = 19.32 | F (1, 18) = 22.64 | P = 0.4600 | P = 0.0003 | P = 0.0002 |
| CD200R | F (1, 20) = 5.619 | F (1, 20) = 50.32 | F (1, 20) = 23.82 | P = 0.0279 | P < 0.0001 | P < 0.0001 |
| CX3CR1 | F (1, 20) = 0.02866 | F (1, 20) = 19.9 | F (1, 20) = 1.2 | P = 0.8673 | P = 0.0002 | P = 0.2864 |
| DAP12 | F (1, 16) = 15.62 | F (1, 16) = 88.15 | F (1, 16) = 213.5 | P = 0.0011 | P < 0.0001 | P < 0.0001 |
| DR3 | F (1, 20) = 17.8 | F (1, 20) = 28.44 | F (1, 20) = 21.89 | P = 0.0004 | P < 0.0001 | P = 0.0001 |
| HMGB1 | F (1, 20) = 5.591 | F (1, 20) = 2.814 | F (1, 20) = 2.304 | P = 0.0283 | P = 0.1090 | P = 0.1447 |
| IKKβ | F (1, 20) = 5.945 | F (1, 20) = 133.5 | F (1, 20) = 4.444 | P = 0.0242 | P < 0.0001 | P = 0.0478 |
| IL-10 | F (1, 18) = 7.441 | F (1, 18) = 27.28 | F (1, 18) = 6.116 | P = 0.0138 | P < 0.0001 | P = 0.0236 |
| IL-13R | F (1, 17) = 5.141 | F (1, 17) = 55.98 | F (1, 17) = 14.15 | P = 0.0367 | P < 0.0001 | P = 0.0016 |
| IL-1β | F (1, 17) = 11.56 | F (1, 17) = 9.958 | F (1, 17) = 9.796 | P = 0.0034 | P = 0.0058 | P = 0.0061 |
| IL-4 | F (1, 18) = 8.579 | F (1, 18) = 6.966 | F (1, 18) = 7.87 | P = 0.0090 | P = 0.0167 | P = 0.0117 |
| IL-4R | F (1, 20) = 26.6 | F (1, 20) = 93.83 | F (1, 20) = 22.62 | P < 0.0001 | P < 0.0001 | P = 0.0001 |
| iNOS | F (1, 20) = 8.366 | F (1, 20) = 2.432 | F (1, 20) = 17.36 | P = 0.0090 | P = 0.1346 | P = 0.0005 |
| MCP1 | F (1, 20) = 0.0009873 | F (1, 20) = 10.39 | F (1, 20) = 8.248 | P = 0.9752 | P = 0.0043 | P = 0.0094 |
| MD-2 | F (1, 18) = 2.641 | F (1, 18) = 9.125 | F (1, 18) = 0.8152 | P = 0.1215 | P = 0.0073 | P = 0.3785 |
| RAGE | F (1, 20) = 0.0826 | F (1, 20) = 50.32 | F (1, 20) = 0.05838 | P = 0.7768 | P < 0.0001 | P = 0.8115 |
| TBK1 | F (1, 19) = 0.1012 | F (1, 19) = 139.2 | F (1, 19) = 0.1911 | P = 0.7539 | P < 0.0001 | P = 0.6670 |
| TGFβ | F (1, 20) = 5.726 | F (1, 20) = 57.65 | F (1, 20) = 4.459 | P = 0.0266 | P < 0.0001 | P = 0.0475 |
| TLR3 | F (1, 17) = 1.617 | F (1, 17) = 8.297 | F (1, 17) = 2.516 | P = 0.2206 | P = 0.0104 | P = 0.1311 |
| TLR4 | F (1, 20) = 0.003965 | F (1, 20) = 227.6 | F (1, 20) = 0.02322 | P = 0.9504 | P < 0.0001 | P = 0.8804 |
| TLR7 | F (1, 18) = 11.27 | F (1, 18) = 21.28 | F (1, 18) = 1.27 | P = 0.0035 | P = 0.0002 | P = 0.2745 |
| TNFα | F (1, 19) = 34.6 | F (1, 19) = 0.824 | F (1, 19) = 13.45 | P < 0.0001 | P = 0.3754 | P = 0.0016 |
| TREM1 | F (1, 16) = 8.257 | F (1, 16) = 27.62 | F (1, 16) = 8.928 | P = 0.0110 | P < 0.0001 | P = 0.0087 |
| TREM2 | F (1, 17) = 0.001245 | F (1, 17) = 1.828 | F (1, 17) = 16.02 | P = 0.9723 | P = 0.1941 | P = 0.0009 |

**Supplementary Table S3.** Post-hoc Bonferroni analyses of SH-SY5Y neuroblastoma +/- BV2 microglia co-culture.

| **9** | **Alone CON**  **vs.**  **Alone EtOH**  **P-Value (Bonferroni)** | **Alone CON**  **vs.**  **Co-Cultured CON**  **P-Value (Bonferroni)** | **Alone EtOH**  **vs.**  **Co-Cultured EtOH**  **P-Value (Bonferroni)** | **Co-Cultured CON**  **vs.**  **Co-Cultured EtOH**  **P-Value (Bonferroni)** |
| --- | --- | --- | --- | --- |
| ADAM10 | >0.9999 | 0.0012** | <0.0001 | 0.0001 |
| Caspase 3 | >0.9999 | 0.0031** | 0.0095 | >0.9999 |
| CCR2 | 0.306 | >0.9999 | >0.9999 | >0.9999 |
| CD200 | >0.9999 | 0.0113* | <0.0001 | 0.0001 |
| ChAT | >0.9999 | 0.6354 | 0.446 | >0.9999 |
| CX3CL1 | 0.8831 | <0.0001**** | <0.0001 | 0.0054 |
| CXCL10 | >0.9999 | 0.0348* | >0.9999 | 0.0131 |
| DAP12 | >0.9999 | 0.0004*** | 0.0011 | >0.9999 |
| DCX | >0.9999 | <0.0001**** | <0.0001 | >0.9999 |
| DR3 | >0.9999 | <0.0001**** | <0.0001 | 0.2187 |
| FADD | >0.9999 | 0.8644 | 0.0011 | 0.0178 |
| Fas | 0.4463 | 0.1053 | 0.0124 | 0.0841 |
| HDAC1 | >0.9999 | 0.8371 | 0.0032 | 0.0756 |
| HDAC2 | >0.9999 | 0.0176* | 0.1704 | >0.9999 |
| HMGB1 | 0.8919 | 0.053 | 0.755 | 0.0433 |
| HMOX1 | <0.0001**** | >0.9999 | 0.0001 | >0.9999 |
| IKKβ | 0.035* | 0.3563 | 0.0021 | 0.0002 |
| IL-10 | >0.9999 | 0.1977 | 0.7455 | >0.9999 |
| IL-13R | >0.9999 | <0.0001**** | <0.0001 | <0.0001 |
| IL-4R | 0.0014* | 0.3047 | <0.0001 | <0.0001 |
| iNOS | 0.0229* | 0.1129 | >0.9999 | >0.9999 |
| MCP1 | 0.2308 | 0.0369* | >0.9999 | 0.0049 |
| NGFR | <0.0001**** | >0.9999 | <0.0001 | >0.9999 |
| RAGE | >0.9999 | <0.0001**** | 0.0004*** | >0.9999 |
| TBK1 | >0.9999 | 0.713 | 0.0005*** | 0.0012** |
| TGFβ | >0.9999 | 0.0005*** | <0.0001**** | <0.0001**** |
| TH | >0.9999 | <0.0001**** | >0.9999 | <0.0001**** |
| TL1A | >0.9999 | <0.0001**** | 0.0456* | 0.0045** |
| TLR3 | 0.0125* | >0.9999 | 0.0023** | >0.9999 |
| TLR4 | >0.9999 | 0.2753 | 0.0002*** | 0.0035** |
| TLR7 | 0.0105* | >0.9999 | 0.0034** | >0.9999 |
| TNFα | >0.9999 | >0.9999 | >0.9999 | >0.9999 |
| TREM1 | >0.9999 | >0.9999 | 0.0009*** | 0.025* |
| TREM2 | >0.9999 | >0.9999 | >0.9999 | 0.4933 |

**Supplementary Table S4.** Table of statistics (F-values, *p*-values) for SH-SY5Y neuroblastoma co-culture data.

|  | **F-values** | | | **P-values** | | |
| --- | --- | --- | --- | --- | --- | --- |
| **Gene** | **Interaction (Co-Culture × Ethanol)** | **Effect of Co-Culture** | **Effect of Ethanol** | **Interaction (Co-Culture × Ethanol)** | **Effect of Co-Culture** | **Effect of Ethanol** |
| ADAM10 | F (1, 20) = 11.47 | F (1, 20) = 96.59 | F (1, 20) = 19.77 | P = 0.0029 | P < 0.0001 | P = 0.0002 |
| Caspase 3 | F (1, 19) = 0.04712 | F (1, 19) = 30.74 | F (1, 19) = 2.977 | P = 0.8305 | P < 0.0001 | P = 0.1007 |
| CCR2 | F (1, 20) = 0.5224 | F (1, 20) = 0.6334 | F (1, 20) = 4.899 | P = 0.4782 | P = 0.4354 | P = 0.0387 |
| CD200 | F (1, 18) = 13.42 | F (1, 18) = 73.38 | F (1, 18) = 19.69 | P = 0.0018 | P < 0.0001 | P = 0.0003 |
| ChAT | F (1, 19) = 0.03815 | F (1, 19) = 6.443 | F (1, 19) = 2.36 | P = 0.8472 | P = 0.0201 | P = 0.1409 |
| CX3CL1 | F (1, 19) = 3.245 | F (1, 19) = 150.3 | F (1, 19) = 15.11 | P = 0.0875 | P < 0.0001 | P = 0.0010 |
| CXCL10 | F (1, 20) = 4.695 | F (1, 20) = 4.839 | F (1, 20) = 7.856 | P = 0.0425 | P = 0.0397 | P = 0.0110 |
| DAP12 | F (1, 19) = 0.2582 | F (1, 19) = 47.31 | F (1, 19) = 0.2751 | P = 0.6172 | P < 0.0001 | P = 0.6060 |
| DCX | F (1, 19) = 0.005019 | F (1, 19) = 242.4 | F (1, 19) = 0.0166 | P = 0.9443 | P < 0.0001 | P = 0.8988 |
| DR3 | F (1, 20) = 0.5018 | F (1, 20) = 122.7 | F (1, 20) = 6.063 | P = 0.4869 | P < 0.0001 | P = 0.0230 |
| FADD | F (1, 19) = 4.402 | F (1, 19) = 18.52 | F (1, 19) = 8.016 | P = 0.0495 | P = 0.0004 | P = 0.0107 |
| Fas | F (1, 16) = 0.5893 | F (1, 16) = 20.01 | F (1, 16) = 11.06 | P = 0.4539 | P = 0.0004 | P = 0.0043 |
| HDAC1 | F (1, 18) = 2.729 | F (1, 18) = 15.65 | F (1, 18) = 6.142 | P=0.1159 | P = 0.0009 | P = 0.0233 |
| HDAC2 | F (1, 19) = 0.4048 | F (1, 19) = 16.56 | F (1, 19) = 1.176 | P = 0.5322 | P = 0.0007 | P = 0.2917 |
| HMGB1 | F (1, 19) = 10.34 | F (1, 19) = 1.013 | F (1, 19) = 1.296 | P = 0.0045 | P = 0.3269 | P = 0.2690 |
| HMOX1 | F (1, 20) = 17.92 | F (1, 20) = 13.77 | F (1, 20) = 29.76 | P = 0.0004 | P = 0.0014 | P < 0.0001 |
| IKKβ | F (1, 19) = 3.075 | F (1, 19) = 20.44 | F (1, 19) = 36.46 | P = 0.0956 | P = 0.0002 | P < 0.0001 |
| IL-10 | F (1, 17) = 0.1913 | F (1, 17) = 7.692 | F (1, 17) = 2.711 | P = 0.6674 | P = 0.0130 | P = 0.1180 |
| IL-13R | F (1, 13) = 101.2 | F (1, 13) = 563.9 | F (1, 13) = 107.5 | P < 0.0001 | P < 0.0001 | P < 0.0001 |
| IL-4R | F (1, 20) = 16.7 | F (1, 20) = 49.36 | F (1, 20) = 108.3 | P = 0.0006 | P < 0.0001 | P < 0.0001 |
| iNOS | F (1, 20) = 3.267 | F (1, 20) = 3.267 | F (1, 20) = 7.951 | P = 0.0857 | P = 0.0857 | P = 0.0106 |
| MCP1 | F (1, 20) = 1.478 | F (1, 20) = 9.706 | F (1, 20) = 18.91 | P = 0.2383 | P = 0.0054 | P = 0.0003 |
| NGFR | F (1, 19) = 89.92 | F (1, 19) = 94.38 | F (1, 19) = 113.2 | P < 0.0001 | P < 0.0001 | P < 0.0001 |
| RAGE | F (1, 17) = 0.8617 | F (1, 17) = 65.24 | F (1, 17) = 0.5731 | P = 0.3663 | P < 0.0001 | P = 0.4594 |
| TBK1 | F (1, 19) = 6.188 | F (1, 19) = 22.49 | F (1, 19) = 14.67 | P = 0.0223 | P = 0.0001 | P = 0.0011 |
| TGFβ | F (1, 20) = 43.97 | F (1, 20) = 185.3 | F (1, 20) = 72.22 | P < 0.0001 | P < 0.0001 | P < 0.0001 |
| TH | F (1, 18) = 19.1 | F (1, 18) = 17.25 | F (1, 18) = 16.08 | P = 0.0004 | P = 0.0006 | P = 0.0008 |
| TL1A | F (1, 20) = 7.885 | F (1, 20) = 49.07 | F (1, 20) = 7.885 | P = 0.0109 | P < 0.0001 | P = 0.0109 |
| TLR3 | F (1, 16) = 5.431 | F (1, 16) = 15.92 | F (1, 16) = 8.157 | P = 0.0332 | P = 0.0011 | P = 0.0114 |
| TLR4 | F (1, 18) = 5.858 | F (1, 18) = 29.74 | F (1, 18) = 12 | P = 0.0263 | P < 0.0001 | P = 0.0028 |
| TLR7 | F (1, 17) = 12.61 | F (1, 17) = 3.872 | F (1, 17) = 3.435 | P = 0.0025 | P = 0.0656 | P = 0.0813 |
| TNFα | F (1, 19) = 0.08961 | F (1, 19) = 0.08961 | F (1, 19) = 1.611 | P = 0.7679 | P = 0.7679 | P = 0.2196 |
| TREM1 | F (1, 18) = 5.106 | F (1, 18) = 16.84 | F (1, 18) = 6.911 | P = 0.0365 | P = 0.0007 | P = 0.0170 |
| TREM2 | F (1, 19) = 0.3617 | F (1, 19) = 0.34 | F (1, 19) = 4.222 | P = 0.5546 | P = 0.5667 | P = 0.0539 |
